# Measurement Invariance of Social Media Use in Younger and Older Adults and Links to Socioemotional Health

**DOI:** 10.1093/geroni/igab009

**Published:** 2021-03-11

**Authors:** Neika Sharifian, A Zarina Kraal, Afsara B Zaheed, Ketlyne Sol, Emily P Morris, Laura B Zahodne

**Affiliations:** Department of Psychology, University of Michigan, Ann Arbor, Michigan, USA

**Keywords:** Age differences, Measurement invariance, Social media use, Socioemotional functioning

## Abstract

**Background and Objectives:**

Social media use has been linked to socioemotional health; however, less is known regarding whether these associations are moderated by age. Additionally, as the use of social media in older adult populations is rapidly increasing, there is a greater need for the investigation of psychometric properties of social media usage scales before determining age differences in the impact of social media on socioemotional health outcomes.

**Research Design and Methods:**

Using an online adult life-span sample (*n* = 592), the current cross-sectional study tested the measurement invariance of the general social media usage subscale of the Media and Technology Usage and Attitudes Scale across younger (aged 19–54) versus older (aged 55–81) adults and whether age moderated associations between social media use and socioemotional health (depressive symptoms, self-esteem, and envy).

**Results:**

Confirmatory factor analyses revealed that posting-related and checking-related items were noninvariant across age groups. In multigroup structural equation models accounting for differential item functioning, higher social media use was associated with more depressive symptoms in younger adults, but not in older adults. While higher social media use was associated with higher envy in both age groups, this association was stronger in younger adults.

**Discussion and Implications:**

Findings suggest younger adults may be more susceptible to the detrimental effects of social media use on socioemotional health. Future directions regarding the measurement of social media use and the salience of social media use across the life span are discussed.

**Translational Significance:** This study found that general items (e.g., checking social media without reference to a specific context) and passive items (e.g., browsing profiles) measured social media use more similarly across younger and older adults. Additionally, the current study found that social media use influenced socioemotional health outcomes to a greater extent in younger adults compared with older adults. These findings may have implications for how future studies measure social media use in older adults as well as how social technology may be utilized in intervention research across the adult life span. If older adults are less affected by the negative consequences of social media use, this may be a potential tool to combat social isolation and loneliness in later life.

Although prior research has investigated the association between social media use and socioemotional health, the nature of these associations is unclear. Some studies report that social media use is linked to better socioemotional outcomes such as higher self-esteem (Gonzales & Hancock, 2011), higher subjective well-being (Nabi et al., 2013), and fewer depressive symptoms (Grieve et al., 2013). Other studies report that higher social media use is linked to worse socioemotional outcomes such as more depressive symptoms (Rosenthal et al., 2016; Shensa et al., 2017), worse mood (Bennett et al., 2020; Sagioglou & Greitemeyer, 2014), and higher envy (Sharifian et al., 2021). As prior research linking social media use and socioemotional health is mixed, an investigation into potential individual differences that moderate this association may help to clarify the extant literature. This study is focused on age, an important individual characteristic that may influence the impact of social media use on socioemotional health.

## The Moderating Role of Age

Social media use may differentially influence socioemotional outcomes across age groups, in part, due to distinct life experiences with technology. Specifically, contemporary younger adults grew up with digital technologies (digital natives), whereas contemporary older adults were introduced to digital technology later in life (digital immigrants; Prensky, 2001). The unique experience of growing up with these technologies may fundamentally change how individuals think and interact with their digital environments (Prensky, 2001). For example, as social media helps to facilitate social relationships and emphasizes self-presentation (e.g., posting photos, status updates), growing up using these media platforms may influence the development of social bonds and identity formation among digital natives (for review, see Shapiro & Margolin, 2014).

Although adults across various life stages have differing experiences with technology, including social media, it is unclear how these differences may influence the impact of social media use on socioemotional outcomes. Scarce research has focused on the link between social media use and socioemotional outcomes in older adults (Francis, 2019; Nam, 2019; Zhou, 2018), and even fewer empirical studies have examined whether the association between social media use and socioemotional health varies across age (Hardy & Castonguay, 2018; Hayes et al., 2015). In a cross-sectional study examining the association between the number of social media platforms used and self-reported “nervous breakdown,” younger adults who reported using more social media platforms had lower risk, whereas for middle-aged and older adults, using more social media platforms was associated with higher risk (Hardy & Castonguay, 2018). Contrastingly, in another cross-sectional study examining psychosocial outcomes of Facebook use across age groups, older age groups (i.e., middle-aged and older adults) evidenced less negative body image on Facebook, less trouble controlling their Facebook use, and less negative social comparison compared with the younger adults (Hayes et al., 2015). Younger adults also reported having more positive fulfillment on Facebook compared with older adults (Hayes et al., 2015), which suggests that younger adults may be more strongly affected by social media use, regardless of whether the outcomes are positive or negative. Overall, the evidence is mixed regarding the impact of age on social media–socioemotional health associations.

## Measurement Invariance of Social Media Use

One potential complication in research examining age group differences in the effects of social media use relates to measurement. If commonly used instruments do not measure social media use similarly across age groups, then age-related differences in the association between social media use and socioemotional health outcomes may be an artifact of measurement noninvariance (i.e., instruments measuring different constructs across groups). As commonly used measures to assess social media use were developed in primarily younger populations, these measures may not be measuring the same construct in older populations.

In particular, the Media and Technology Usage and Attitudes Scale (MTUAS; Rosen et al., 2013) is a commonly used scale that prior research has used to measure social media and technology usage (Rashid & Asghar, 2016; Spradlin et al., 2019). Some prior research has investigated the psychometric properties of the MTUAS in samples from the United States (Rosen et al., 2013), Turkey (Özgür, 2016), and Portugal (Costa et al., 2016). Additionally, prior research has also extended the use of the MTUAS to adolescent populations (Costa et al., 2016). In general, these studies found the MTUAS to be a valid and reliable measure for social media usage across these culturally diverse samples (i.e., see the systematic review of, Sigerson & Cheng, 2018). To the best of our knowledge, however, less is known regarding measurement invariance of the MTUAS outside of adolescent and predominantly young adult populations. Thus, establishing measurement invariance of the social media instrument (i.e., MTUAS) across younger and older adult age groups is necessary before interpreting age moderation of associations between social media use and socioemotional health in an adult life-span sample.

## The Present Study

This study first aimed to test whether the assessment of social media use was invariant across younger and older adult populations. Second, after establishing or correcting for measurement invariance, we aimed to examine associations between social media use and socioemotional health in an age-heterogeneous adult sample and test whether these associations were moderated by age. As prior research regarding the socioemotional impact of social media use (Bennett et al., 2020; Grieve et al., 2013; Nabi et al., 2013; Rosenthal et al., 2016) and the moderating role of age is mixed (Hardy & Castonguay, 2018; Hayes et al., 2015), we had no a priori hypotheses regarding the nature of these effects.

## Method

### Participants and Procedure

Participants were recruited through Amazon’s Mechanical Turk (MTurk) and were restricted to those residing within the United States and those who had demonstrated a 95% approval rating from other completed Human Intelligence Tasks (HITs). Participants were compensated $1.00 for their participation in the survey. As prior research has identified that the MTurk population has a lower proportion of older adults relative to younger adults (Difallah et al., 2018; Hitlin, 2016), half of the sample (*n* = 350) was restricted to the MTurk 55 years or older premium qualification to ensure an adult life-span sample. All data collection was completed in September 2019. All participants provided informed consent, and all study procedures were approved by the University of Michigan’s institutional review board.

As described in previous research (Sharifian et al., 2021), 706 participants completed the survey on MTurk. Of those participants, 114 were excluded because of at least one of the following data quality issues: (a) a mismatch between chronological age and birthdate (*n* = 74), (b) self-reported engagement in other activities like other surveys/HITs during the survey (*n* = 15), and (c) survey completion time 2 *SD*s above or below the sample average (*n* = 25). The final sample included 592 adults ranging from 19 to 81 years of age (*M*_age_ = 50.63, *SD*_age_ = 15.89, 58.40% female). Participants took, on average, 18.73 min to complete the survey (*SD* = 6.68 min).

### Measures

*Age* was self-reported as a continuous variable and confirmed via self-reported birthdate. In order to test our research questions, age was dichotomized into two groups. Age groups were determined based on the bimodal distribution of age within our sample (Supplementary Figure 1) and previous gerontological research using samples aged 55 and older (Ailshire & Clarke, 2015; Weller et al., 2014). Participants who reported being 19 to 54 years old were categorized as younger adults (*n* = 258, 43.60%), and participants who reported being 55 to 81 years old were categorized as older adults (*n* = 334, 56.40%).

*Social media use* was assessed using the general social media usage subscale of the MTUAS (Rosen et al., 2013). Participants were asked how often they engaged in the following activities on social networking sites: (1) check your social networking sites such as Facebook and Instagram, (2) check your social networking sites such as Facebook and Instagram from your smartphone, (3) check social networking sites such as Facebook and Instagram at work or school, (4) post status updates, (5) post photos, (6) browse profiles and photos, (7) read postings, (8) comment on postings, status updates, photos, etc., and (9) click “Like” to a posting, photo, etc. All items were rated on a 10-point scale ranging from 1 (*never*) to 10 (*all the time*) and were included as observed indicators of a latent social media use variable with higher scores representing higher social media use. The scale demonstrated good internal consistency in the overall sample (α = 0.94).

*Socioemotional functioning* was assessed using three measures: depressive symptoms, self-esteem, and envy. Depressive symptoms over the past week were measured using an eight-item version of the Center for Epidemiologic Studies—Depression scale (Radloff, 1977). Participants rated items such as “I felt depressed” on a 4-point Likert scale ranging from 1 [*Rarely or none of the time (less than 1 day)*] to 4 [*Most or all of the time (5–7 days)*], so higher scores correspond to more depressive symptoms. An average across the eight items was computed and demonstrated good internal consistency in the overall sample (α = 0.90).

Self-esteem was measured using a 10-item self-esteem questionnaire (Rosenberg, 1965). Items such as “On the whole, I am satisfied with myself” (reverse-coded) were rated on a 4-point scale ranging from 1 (*strongly agree*) to 4 (*strongly disagree*). Items were coded such that higher scores represented higher self-esteem. An average across the eight items was computed and demonstrated good internal consistency in the overall sample (α = 0.92).

Envy was measured with the eight-item dispositional envy scale (Smith et al., 1999). Participants rated responses to items such as “The bitter truth is that I generally feel inferior to others” using a 5-point scale ranging from 1 (*strongly disagree*) to 5 (*strongly agree*), so higher scores correspond to greater envy. An average across the eight items was computed and demonstrated good internal consistency in the overall sample (α = 0.95).

#### Covariates

The following covariates were included in the age moderation analyses: age, gender, education, and self-rated health. To control for heterogeneity of age within each age group, we included self-reported age as a covariate in our analyses, which was represented as a continuous variable. Gender was represented by a dichotomous variable (0 = Male and 1 = Female). Education was assessed with a single item asking participants to self-report the highest grade of school they completed ranging from 1 (*No school/some grade* school) to 12 (*PhD, EDD, MD, DDS, LLB, LLD, JD, or other professional degrees*) and was included as a continuous variable. Self-rated health was assessed with a single item asking participants to rate their overall physical health on a 7-point scale ranging from 1 (*poor*) to 7 (*excellent*).

### Analytic Strategy

In order to examine measurement invariance of the general social media usage scale across age groups (Aim 1), four sequential steps were conducted according to recommendations for assessing configural, metric, scalar, and residual invariance (Putnick & Bornstein, 2016). First, a confirmatory factor analysis (CFA) was conducted to assess *configural invariance*, defined as support for the same basic factor structure (i.e., one underlying factor) in both groups. In the second step, a CFA was conducted assessing *metric invariance*, which is defined as the equivalence of item factor loadings and is tested by constraining the factor loadings to be equivalent across groups. If metric noninvariance was found, we investigated sources of noninvariance by examining modification indices. Using the backward method (sequentially releasing constraints) and starting with the parameter with the largest modification index, constraints were sequentially released until partial measurement invariance was achieved. In the third step, we conducted a CFA assessing *scalar invariance*, which is defined as the equivalence of the item intercepts (for metric-invariant items) and is tested by constraining the item intercepts to be equivalent across groups. Similar to the previous step, if noninvariance was found, we tested for sources of noninvariance until partial scalar invariance was achieved. Finally, a fourth CFA was conducted to test for *residual invariance*, which is defined as the equivalence of item residuals (for metric- and scalar-invariant items) and is tested by constraining the residual item variances to be equivalent across groups. Subsequently, we tested whether groups differed in the underlying construct of social media use, adjusting for any measurement invariance detected through the procedures described above and below.

Model fit was compared with each preceding model in order to determine support for each category of measurement invariance. For instance, metric invariance is supported if the overall model fit in the metric invariance CFA model is not significantly worse in comparison to the configural invariance model and so forth at each step. As prior research has suggested that chi-square difference tests are overly sensitive to detecting noninvariance, we used the following criteria to determine measurement invariance at each step: (a) lowering of the comparative fit index (CFI) by no more than 0.01 (Chen, 2007; Cheung & Rensvold, 2002), (b) a change in root mean square error of approximation (RMSEA) less than 0.015, and (c) a change in standardized root mean square residual (SRMR) less than 0.030 for metric invariance and 0.015 for scalar or residual invariance (Chen, 2007).

In order to test for age moderation of associations between social media use and socioemotional outcomes (Aim 2), we conducted multigroup modeling. Specifically, socioemotional outcomes (depressive symptoms, self-esteem, and envy) were regressed onto the latent social media use variable. Covariates were controlled for on the exposure and outcomes. Separate models were conducted for each socioemotional domain. In a series of nested multigroup models, the path between social media use and each socioemotional measure was initially constrained and then subsequently allowed to vary across age groups. Chi-square differences between constrained and freed models were compared to assess whether significant age moderation was present. All analyses were conducted in Mplus (version 8.5).

If measurement noninvariance was identified through Aim 1, we conducted the following analyses to assess the robustness of findings regarding age moderation. First, as recommended by Chen (2008), we ran our multigroup model allowing for partial invariance by imposing constraints on only invariant items and allowing noninvariant items to vary across age groups. Next, we ran models assuming full measurement invariance, in which we imposed constraints across all items, regardless of whether they were found to be invariant or noninvariant.

Second, we conducted a robustness technique in which we ran a reduced (noninvariant items removed) fully invariant model (Cheung & Rensvold, 1998) and compared the resultant substantive conclusions (i.e., a pattern of major findings) to those from the partial invariance models. If the findings were similar across the models (i.e., partial vs. full; partial vs. reduced), we concluded that measurement noninvariance had little impact on the Aim 2 results. If the findings differed substantively across models, then we concluded that measurement noninvariance had a significant impact on the Aim 2 results, warranting caution in the interpretation of age moderation.

## Results

### Measurement Invariance

Descriptive statistics for variables of interest are listed in Table 1. Model fit statistics and changes in model fit statistics that were used to assess measurement invariance are listed in Table 2.

**Table 1.** Descriptive Statistics for Main Variables of Interest

	Full sample (*n* = 592)		Adults aged 19–54 (*n* = 258)		Adults aged 55–81 (*n* = 334)	
Variables	*M*	*SD*	*M*	*SD*	*M*	*SD*
Age (continuous)	50.63	15.89	34.07	7.67	63.43	5.09
% Female	58.40	—	45.00	—	68.90	—
Education (1–12)	8.28	2.05	8.41	2.04	8.17	2.06
Self-reported health (1–7)	4.77	1.47	4.97	1.42	4.62	1.50
Social media use (1–10)	4.94	2.11	5.77	2.13	4.29	1.86
Self-esteem (1–4)	3.12	0.65	2.95	0.65	3.25	0.62
Envy (1–5)	1.93	1.08	2.40	1.21	1.56	0.79
Depressive symptoms (1–4)	1.82	0.73	1.91	0.74	1.75	0.72

**Table 2.** Model Fit Statistics for Investigating Measurement Invariance Across Age Groups

Models		χ ^2^	CFI	RMSEA	SRMR	Comp	Δχ ^2^	ΔCFI	ΔRMSEA	ΔSRMR	Decision
M1	Configural invariance	140.34	0.976	0.103 (0.09–0.12)	0.032	—	—	—	—	—	—
M2	Metric invariance	191.35	0.966	0.110 (0.09–0.13)	0.071	M1	51.01	−0.01	0.007	0.039	Reject
M2a	Partial metric invariance	160.40	0.972	0.101 (0.09–0.12)	0.050	M1	20.07	−0.004	−0.002	0.018	Accept
M3	Scalar invariance	258.12	0.951	0.125 (0.11–0.14)	0.063	M2a	97.71	−0.021	0.024	0.013	Reject
M3a	Partial scalar invariance	188.30	0.967	0.105 (0.09–0.12)	0.049	M2a	27.89	−0.005	0.004	−0.001	Accept
M4	Residual invariance	199.08	0.966	0.102 (0.09–0.12)	0.050	M3a	10.78	−0.001	−0.003	0.001	Accept

*Note:* CFI = comparative fit index; RMSEA = root mean square error of approximation; SRMR = standardized root mean square residual; Comp = model comparison.

The initial factor structure demonstrated adequate model fit, indicative of configural invariance for the one-factor structure of social media use across the two age groups. In the next step, a comparison of model fit statistics showed that there was a difference between the metric and the configural model. To test for partial metric invariance, an identification of the largest modification index led to the decision to initially free the factor loading for item 5 (“post photos”), followed by the factor loading for item 4 (“post status updates”). The item “post photos” showed a higher factor loading for adults aged 19–54 (standardized factor loading = 0.78) compared with adults aged 55–81 (standardized factor loading = 0.69). Similarly, the item “post status updates” showed a higher factor loading for adults aged 19–54 (standardized factor loading = 0.77) compared with adults aged 55–81 (standardized factor loading = 0.69). Subsequent examination of model fit statistics for the partial metric invariance model demonstrated negligible changes in CFI, RMSEA, and SRMR compared with the configural model.

Next, a comparison of model fit statistics for the scalar and the partial metric model showed a change in model fit. We tested for partial scalar invariance through an examination of modification indices. Starting with the largest modification index, the intercept of item 3 (“check social networking sites such as Facebook and Instagram at work or school”) was initially freed across age groups. Subsequently, the intercept of item 2 (“check your social networking sites such as Facebook and Instagram on your smartphone”) was freed across age groups. Above and beyond any differences in underlying social media use, adults aged 19–54 (standardized intercept = −0.44) were more likely to endorse checking social media at work/school than adults aged 55–81 (standardized intercept = −0.91), independent of underlying social media use. Adults aged 19–54 (standardized intercept = −0.49) were also more likely to endorse checking social media on their smartphones than adults aged 55–81 (standardized intercept = −0.77). Subsequent examination of model fit criteria of the partial scalar invariance model demonstrated negligible changes in CFI, RMSEA, and SRMR compared with the partial metric invariance model. Finally, a comparison of model fit statistics for the residual model and the partial scalar invariance model demonstrated negligible differences based on CFI, RMSEA, and SRMR indices.

After accounting for partial measurement invariance, we tested whether the latent mean of social media use differed across age groups by constraining the mean to be equal across groups. This revealed a significant chi-square difference (Δχ ^2^ = 42.26) as well as changes in CFI (ΔCFI = −0.01) and SRMR (ΔSRMR = 0.058), indicating that adults aged 19–54 had significantly higher social media use compared with adults aged 55–81 (6.84 vs. 5.89).

### Age Moderation

For all three socioemotional health measures, we found that model fit significantly improved when freeing the path between social media use and socioemotional health across age groups (Table 3), indicating significant age moderation. Model fit for the depressive symptoms (χ ^2^ (129) = 377.29, CFI = 0.95, RMSEA = 0.08 [0.07–0.09], SRMR = 0.05), self-esteem (χ ^2^ (129) = 365.74, CFI = 0.95, RMSEA = 0.08 [0.07–0.09], SRMR = 0.05), and envy (χ ^2^ (129) = 390.78, CFI = 0.94, RMSEA = 0.08 [0.07–0.09], SRMR = 0.05) was generally adequate. All covariate associations for each model can be found in Supplementary Table 1.

**Table 3.** Chi-Square Differences for Testing Age Moderation in Multigroup Models

	Partially invariant models		Fully invariant models		Reduced fully invariant models	
Models	χ ^2^	Δχ ^2^	χ ^2^	Δχ ^2^	χ ^2^	Δχ ^2^
Constrained path: social media → envy	414.45	—	644.15	—	178.95	—
Constrained path: social media → self-esteem	370.17	—	599.73	—	142.68	—
Constrained path: social media → depressive symptoms	386.34	—	616.10	—	150.30	—
Unconstrained path: social media → envy	390.78	23.67	620.94	23.21	161.78	17.17
Unconstrained path: social media → self-esteem	365.74	4.43	595.25	4.48	139.79	2.89
Unconstrained path: social media → depressive symptoms	377.29	9.05	607.05	9.05	142.73	7.57

*Notes:* Partially measurement invariant models account for measurement noninvariance of the following Media Technology Usage and Attitudes Scale social media use items: post status updates, post photos, check social media on a smartphone, and check social media at work/school. Fully measurement invariant models assume complete measurement invariance as a robustness check, following recommendations by Chen (2008). Reduced fully invariant models only include items that were fully invariant, following recommendations by Cheung and Rensvold (1998).

Higher social media use was associated with more depressive symptoms for adults aged 19–54 (β = 0.25, *SE* = 0.06, *p* < .001), but no association was found for adults aged 55–81 (β = 0.003, *SE* = 0.06, *p* = .960). Although we found significant age moderation of the association between social media and self-esteem, these associations were nonsignificant in both age groups (adults aged 19–54: β = −0.09, *SE* = 0.06, *p* = .099; adults aged 55–81: β = 0.07, *SE* = 0.06, *p* = .181) and are therefore not interpreted. Finally, higher social media use was more strongly associated with higher envy in adults aged 19–54 (β = 0.42, *SE* = 0.05, *p* < .001) than adults aged 55–81 (β = 0.13, *SE* = 0.06, *p* = .025). In other words, when individuals reported higher social media use, adults aged 19–54 reported higher envy to a greater extent than adults aged 55–81.

### Robustness of Findings

As partial measurement invariance was found and accounted for in our age moderation, we tested the robustness of our moderation findings initially by following recommendations by Chen (2008). Substantive conclusions from models that fully constrained all items (regardless of whether invariant or noninvariant across groups) were compared with models that allowed for partial measurement invariance. Model fit for self-esteem (χ ^2^ (143) = 595.25, CFI = 0.90, RMSEA = 0.10 [0.10–0.11], SRMR = 0.11), envy (χ ^2^ (143) = 620.94, CFI = 0.90, RMSEA = 0.11 [0.10–0.12], SRMR = 0.11), and depressive symptoms (χ ^2^ (143) = 607.05, CFI = 0.90, RMSEA = 0.11, [0.10–0.11], SRMR = 0.11) was generally poor. Of note, the results revealed the same pattern of findings across these analyses (Table 3).

Next, we tested the robustness of our findings by conducting a reduced fully invariant model (Cheung & Rensvold, 1998) in which the four noninvariant items were removed from the age moderation models. Model fit across age moderation models for self-esteem (χ ^2^ (55) = 139.79, CFI = 0.97, RMSEA = 0.07 [0.06–0.09], SRMR = 0.04), envy (χ ^2^ (55) = 161.78, CFI = 0.96, RMSEA = 0.08 [0.07–0.10], SRMR = 0.05) and depressive symptoms (χ ^2^ (55) = 142.73, CFI = 0.97, RMSEA = 0.07 [0.06–0.09], SRMR = 0.05) was adequate. Similarly, the results revealed the same pattern of findings for the reduced fully invariant models as the partial invariance and fully invariant models.

Finally, as the current study focused on comparing younger and older adults, a sensitivity analysis excluding those who would be traditionally defined as middle-aged (45–64) was conducted. These analyses restricted the sample to younger adults aged 18–44 and older adults aged 65 years and older. Measurement invariance analyses revealed the same four social media items (i.e., posting- and checking-related items) to be noninvariant across younger and older adults. Also consistent with our primary model, age moderation analyses revealed that younger adults (β = 0.43, *SE* = 0.06, *p* < .001) showed stronger associations between social media use and envy compared to older adults (β = 0.24, *SE* = 0.09, *p* = .008). Younger adults (β = 0.26, *SE* = 0.06, *p* < .001) also showed a significant association between social media use and depressive symptoms whereas no association was found for older adults (β = 0.01, *SE* = 0.09, *p* = .933).

## Discussion

This study aimed to examine whether a commonly used measure of social media use was invariant across younger and older adults and to subsequently test whether age moderated associations between social media use and socioemotional health. Overall, we found evidence for partial measurement invariance of the nine-item general social media usage MTUAS subscale. Accounting for partial measurement invariance, we found that younger adults reported higher social media use than older adults, and higher social media use was more strongly associated with worse socioemotional health in younger adults relative to older adults. These findings may implicate the differential ways in which younger and older adults engage with social media and the relative impact of social media use across the various stages of the adult life course.

### Items Differently Related to Social Media Use Across Age Groups

Of the nine MTUAS items assessing social media usage, several items were found to be noninvariant across age groups. Specifically, the items “post status updates” and “post photos” had different factor loadings in the two age groups such that these two items were more strongly correlated with the other MTUAS items assessing social media use in younger adults compared with older adults. This finding may reflect age-related differences in specific activities on social media. Prior research has shown that older age is associated with fewer behaviors associated with posting on Facebook (Chang et al., 2015; McAndrew & Jeong, 2012), which may be partially driven by older adults’ concerns about privacy on social media (Jung et al., 2017; Xie et al., 2012). Additionally, older adults may use social media more passively to keep up with others rather than as a platform for self-expression. Indeed, prior research has shown that older adults engage in more family activity such as viewing relatives’ photos (McAndrew & Jeong, 2012) and view social media/Facebook as an effective tool for keeping up with the lives of family and friends (Jung et al., 2017). As a result, posting updates and photos may not correlate as highly with other social media behaviors among older adults compared with younger adults.

Additionally, we found that intercepts for items involving checking social networking sites on a smartphone or at work/school differed across age groups. Independent of underlying social media use, younger adults were more likely to endorse items regarding checking social networking sites on their smartphone and at work/school compared with older adults. In other words, age differences in the frequency of these behaviors appeared to be driven by age differences in other factors *other than* social media use. Age differences in checking social networking sites at work/school likely reflect developmental shifts such that older adults are less likely to be working or in school compared with younger adults. Checking social network sites on a smartphone may also be less common among older adults due to the digital divide. Older adults are more likely to be digital immigrants with less access to technologies such as smartphones as well as fewer skills to fully utilize these technologies (Scheerder et al., 2017). Furthermore, age differences in the use of social media platforms may also partially explain this pattern of findings. Specifically, individuals in this study were asked to report on which social media platforms they used. Younger adults were more likely to use Instagram, Snapchat, and WhatsApp compared with older adults (Supplementary Table 2). These social media platforms are more commonly used on smartphone devices and, therefore, checking-related behavior may be partially driven by age-related differences in platform usage.

Overall, we found that more general items, such as checking social networking sites without reference to a specific context or device, as well as more passive items (i.e., browsing profiles, reading posts) were more likely to measure social media use similarly across younger and older adults. In contrast, specific items related to actively posting on social media and using social media at work/school or on a smartphone demonstrated measurement noninvariance across younger and older adults, suggesting that these items do not assess social media use in the same way in both groups. Future research should consider potential measurement biases when assessing social media use with these items by accounting for partial measurement invariance of the scale. Although it is notable that the overall pattern of findings was similar regardless of whether measurement noninvariance was taken into account, model fit improved in the reduced fully measurement invariant models compared with the partial measurement invariant and fully measurement invariant models. Therefore, future researchers should consider the removal of these four noninvariant items (i.e., post photos, post status updates, check social networking sites at work or school, and check your social networking sites from your smartphone) when examining social media use with the MTUAS in older adult populations.

On the whole, these results indicate that future aging research should utilize social media usage scales that measure more general and passive behaviors to better capture social media usage in older adult populations. As an alternative to removing the identified noninvariant items, future research could investigate whether adapting these context-specific social media items could adequately capture social media use in older adult populations. For instance, as a lower proportion of older adults reporting owning a smartphone (Scheerder et al., 2017), future research should assess whether adapting the item, “check your social networking sites such as Facebook and Instagram from your smartphone” to a broader context (e.g., on a personal computer or device such as a smartphone or tablet) may better capture older adult social media usage. Similarly, future research should assess whether adapting the item, “check social networking sites such as Facebook and Instagram at work or school” to more general contexts that include post-retirement activities (e.g., volunteering, caregiving, and engaged in other activities) may better capture older adults’ social media usage.

### Social Media Use and Socioemotional Development: Cohort Versus Age Effects

After accounting for partial measurement invariance, we found that younger adults used social media to a greater extent than older adults, consistent with prior evidence that younger adults are more likely to use social media sites (Smith & Anderson, 2018) and use social media sites more frequently (Hayes et al., 2015) compared with older adults. Indeed, in a survey of U.S. teens, 45% of teens reported that they were online almost constantly (Anderson & Jiang, 2018). Furthermore, not only did younger adults use social media more than older adults in this study, they were at greater risk of the negative socioemotional consequences of social media use. Specifically, this study found that younger adults’ use of social media was more strongly associated with worse socioemotional health (i.e., depressive symptoms, envy) compared with older adults’ use.

Because of the cross-sectional design of this study, these findings may reflect cohort differences and/or true age differences. That is, cohort differences may partially explain the greater impact of social media use on socioemotional health in contemporary younger adults due to the role that these social networking sites have in this younger generation’s socioemotional development. In the twenty-first century, younger adults are more likely to be digital natives and to have grown up with the rise of social media, which may have fundamentally changed how they formed their identities and fostered social relationships in adolescence and subsequently into adulthood (see reviews in, Shapiro & Margolin, 2014; Wood et al., 2016).

Younger adults may use these social media to explore and develop their sense of identity by using embedded functions such as posting status updates, pictures, and/or comments. These online behaviors are in line with Erikson’s stage of psychosocial development model (Erikson, 1950), which suggests that younger individuals attempt to form their identity by presenting themselves to others and subsequently modifying their identity based on the reactions from others. Indeed, prior research has shown a robust association between peer relationships and identity formation in adolescence (Meeus et al., 2002). Consistent with our findings and in line with this argumentation, prior research has shown that younger adults are more strongly emotionally affected by social media use compared with older adults (Hayes et al., 2015).

In contrast, contemporary older adults are more likely to be digital immigrants and have adopted social media much later in life and may be more likely to have a more solidified identity before they began to explore the use of social media. Furthermore, prior research has shown that older age is associated with fewer total Facebook friends, but a greater proportion of actual friends on Facebook (i.e., people with shared personal history and meaningful connections; Chang et al., 2015). These findings suggest that older adults use social media to connect to already-established offline relationships, whereas younger adults may include more peripheral members in their online networks which may, in turn, lead to worse socioemotional health (Chang et al., 2015).

Alternatively, age-related changes in socioemotional development that occur in older adulthood may explain age differences in the impact of social media on socioemotional health. Stemming from socioemotional selectivity theory (Carstensen et al., 1999), older adults are theorized to show a preference toward positive relative to negative information as a way to maintain their present-focused goals related to emotional meaning and satisfaction. In contrast, younger adults are theorized to focus on more knowledge-focused goals and have previously been shown to demonstrate the opposite pattern such that younger adults tend to show a preference toward negative information (Reed et al., 2014). It is also theorized that coping strategies such as situation selection and situation modification may be more effective for older adults as a way to maintain emotion-focused goals in comparison to younger adults (Urry & Gross, 2010). As social media sites are user-driven, older adults may intentionally shift their attention toward more positive content as well as selectively choose to follow individuals who post more positive content relative to younger adults.

Overall, it may be the case that age group differences in the impact of social media use on socioemotional health are driven by both cohort effects and aging effects involving socioemotional development at different stages of the life course. Cohort effects may relate to the developmental period during which social technologies were introduced to individuals’ daily lives, while aging effects may relate to developmental changes in socioemotional functioning. Additional research is necessary to further explore how historical and developmental processes may influence the importance and impact of social media use on socioemotional health across the life course. As the use of new social technologies is not consistently experienced across generations (Antonucci et al., 2017), this provides a unique opportunity for future research to examine the associations between social media use and socioemotional health as digital natives transition into older adulthood to help disentangle cohort or true age effects.

### Limitations and Future Directions

Although the current study has several strengths, such as the inclusion of a rigorous test of measurement invariance using multiple indicators to ensure unbiased age comparisons, the use of an age-heterogeneous sample, the inclusion of multiple indicators of socioemotional health to reduce mono-method bias, and direct tests of age moderation, there are several limitations that should be considered.

First, the current study is cross-sectional and as such, caution is warranted in interpreting the directionality of our findings. It may be the case that those with worse socioemotional health use social media to a greater extent or that younger adults use social media to cope with poorer socioemotional health to a greater extent than older adults. Future research should utilize longitudinal and experimental designs to further clarify these associations. Second, as younger and older adults may engage with different social media platforms, future research should use finer-grained assessment of social media usage across platforms (e.g., posting on Facebook vs. Twitter). Third, due to sample size limitations and the bimodal distribution of age of the current study, this study compared younger adults aged 19–54 and older adults aged 55–81. Future research should extend these findings by further examining distinctions between young, middle-aged, and older adults. Of note, contemporary middle-aged adults may be particularly heterogeneous in terms of whether individuals are “digital immigrants” versus “digital natives.” Fourth, although we found support for the harmful effects of social media use for socioemotional health, we did not investigate behavioral factors that may influence how social media use affects socioemotional health. Prior research suggests that several factors, such as the way in which individuals engage with social media (Escobar-Viera et al., 2018), may determine whether social media use results in socioemotional benefits or consequences.

Finally, although our sample was collected online using MTurk, which has previously been validated for survey research (Behrend et al., 2011; Buhrmester et al., 2011), future research should replicate our findings in more nationally representative and community-based in-person samples. Older adults who use MTurk may be more tech-savvy and may be fundamentally different from the typical older adult population due to the gray digital divide, which could limit the generalizability of the current results. Indeed, some evidence comparing older adults on MTurk and older adults from the Health and Retirement Study (HRS) found that older adults recruited from MTurk were younger, more educated, wealthier, and more likely to be female (Ogletree & Katz, 2020). Additionally, MTurk older adults were more likely to have higher performance on verbal analogies and verbal fluency than HRS older adults which was partly attributable to these sociodemographic differences (Ogletree & Katz, 2020). Therefore, the current results may not be generalizable to the larger older adult population.

## Conclusions

We found that the nine-item general social media usage subscale of the MTUAS demonstrated partial measurement invariance, such that items related to posting behaviors and items associated with use at work/school or on a smartphone assessed social media use differently across younger adults and older adults. While statistically accounting for measurement invariance did not substantially alter our conclusions about age differences in social media use or its effects on socioemotional health, future researchers should consider potential measurement bias when examining age differences involving social media use by explicitly modeling measurement bias, removing noninvariant items, or testing the appropriateness of adapted items. For example, future research should investigate whether adapting context-specific items (i.e., “… on a smartphone” or “… at work/school”) to contexts that are more inclusive to older adults may better capture social media use in older adult populations.

These findings also indicated that younger adults may be more likely to experience negative socioemotional consequences from frequent social media use not only because they are more frequent social media users, but also because their socioemotional health appears to be more strongly affected by social media use relative to older adults. These age group differences may reflect changes in socioemotional development for individuals who grew up embedded in these social technologies compared with those who adopted them later in life. Older adults may also be less susceptible to the detrimental effects of social media due to age-related changes in emotional goals and emotion regulation.

## Supplementary Material

igab009_suppl_Supplementary_Materials
