# Supplementary material for: Measurement Invariance of Social Media Use in Younger and Older Adults and Links to Socioemotional Health
Source: Innov Aging. 2021 Mar 11;5(2):igab009. doi: 10.1093/geroni/igab009 (PMC8057130; doi:10.1093/geroni/igab009)
Supplement: igab009_suppl_Supplementary_Materials [file igab009_suppl_supplementary_materials.docx]

**Online Supplementary Material for Publication in *Innovation in Aging*:**

Measurement Invariance of Social Media Use in Younger and Older Adults and Links to Socioemotional Health

Neika Sharifian, PhD, A. Zarina Kraal, PhD, Afsara B. Zaheed, MS, Ketlyne Sol, PhD, Emily P. Morris, MS & Laura B. Zahodne PhD

Department of Psychology, University of Michigan, Ann Arbor, Michigan, USA

*Address correspondence to: Neika Sharifian, PhD, Department of Psychology, University of Michigan, 530 Church Street, Ann Arbor, MI 48109 USA. E-mail: [nsharifi@umich.edu](mailto:nsharifi@umich.edu)

Supplemental Table 1

*Standardized Covariate Associations for Age Moderation Models*

|  | Depressive Symptoms | |  | Self-Esteem | |  | Envy | |
| --- | --- | --- | --- | --- | --- | --- | --- | --- |
| **Adults Aged 19-54** |  |  |  |  |  |  |  |  |
| Age 🡪 DV | -.24 | *** |  | .26 | *** |  | -.23 | *** |
| Female 🡪DV | .00 |  |  | -.06 |  |  | -.01 |  |
| Education 🡪DV | .29 | *** |  | -.31 | *** |  | .28 | *** |
| Health 🡪DV | -.31 | *** |  | .47 | *** |  | -.21 | *** |
| Age 🡪 Social Media | -.18 | ** |  | -.18 | ** |  | -.18 | ** |
| Female 🡪 Social Media | .05 |  |  | .05 |  |  | .05 |  |
| Education 🡪 Social Media | .05 |  |  | .05 |  |  | .05 |  |
| Health 🡪 Social Media | .14 | * |  | .14 | * |  | .14 | * |
| **Adults Aged 55-81** |  |  |  |  |  |  |  |  |
| Age 🡪 DV | -.08 |  |  | .10 |  |  | -.03 |  |
| Female 🡪DV | .06 |  |  | -.01 |  |  | .02 |  |
| Education 🡪DV | -.01 |  |  | -.04 |  |  | -.04 |  |
| Health 🡪DV | -.33 | *** |  | .31 | *** |  | -.22 | *** |
| Age 🡪 Social Media | -.13 | * |  | -.12 | * |  | -.13 | * |
| Female 🡪 Social Media | .19 | ** |  | .18 | * |  | .19 | ** |
| Education 🡪 Social Media | -.10 |  |  | -.10 |  |  | -.10 |  |
| Health 🡪 Social Media | .10 |  |  | .10 |  |  | .10 |  |

*Note*. DV = Dependent Variable, * = p < .05, ** = p < .01, *** = p < .001

Supplemental Table 2

Age Differences in Platform Use

| Platform | Younger Adults aged 19-54 | Older Adults Aged 55+ | Differences |
| --- | --- | --- | --- |
| Facebook | 88.40% | 87.40% | YA = OA |
| Instagram | 65.50% | 35.30% | YA > OA |
| Twitter | 50.80% | 44.60% | YA = OA |
| Tumblr | 4.70% | 7.20% | YA = OA |
| Myspace | 1.20% | 0.60% | YA = OA |
| Snapchat | 22.10% | 4.50% | YA > OA |
| YouTube | 71.70% | 69.20% | YA = OA |
| WhatsApp | 24.00% | 9.30% | YA > OA |
| LinkedIn | 29.10% | 32.60% | YA = OA |
| Reddit | 6.60% | 4.20% | YA = OA |
| Pinterest | 1.20% | 3.00% | YA = OA |
| Other | 1.60% | 3.30% | YA = OA |

*Note*. Binary logistic regressions were conducted to compare age differences in platform use and these findings are listed in the Differences column. Less than or greater than symbols indicate significant differences whereas equal signs indicate nonsignificant differences.


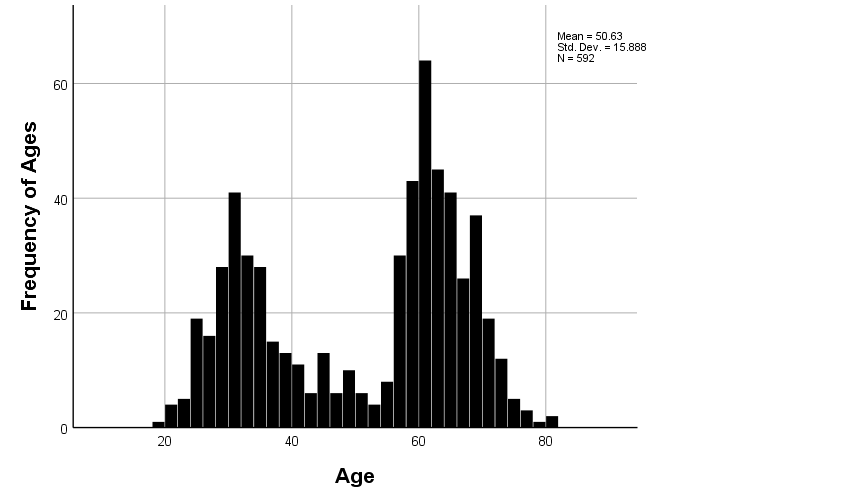


Supplemental Figure 1

Histogram of the Bimodal Frequency Distribution of Age
